# Short‐term mortality prediction in acute pulmonary embolism: Radiomics values of skeletal muscle and intramuscular adipose tissue

**DOI:** 10.1002/jcsm.13488

**Published:** 2024-06-10

**Authors:** Iram Shahzadi, Alex Zwanenburg, Lynn Johann Frohwein, Dominik Schramm, Hans Jonas Meyer, Mattes Hinnerichs, Christoph Moenninghoff, Julius Henning Niehoff, Jan Robert Kroeger, Jan Borggrefe, Alexey Surov

**Affiliations:** ^1^ Department of Radiology, Neuroradiology and Nuclear Medicine Johannes Wesling University Hospital, Ruhr University Bochum Bochum Germany; ^2^ Siemens Healthineers GmbH Erlangen Germany; ^3^ OncoRay‐National Center for Radiation Research in Oncology, Faculty of Medicine, and University Hospital Carl Gustav Carus Technische Universität Dresden, Helmholtz‐Zentrum Dresden‐Rossendorf Dresden Germany; ^4^ National Center for Tumor Diseases (NCT), Partner Site Dresden Dresden Germany; ^5^ Department of Radiology University of Halle Halle Germany; ^6^ Department of Radiology University of Leipzig Leipzig Germany; ^7^ Department of Radiology University of Magdeburg Magdeburg Germany

**Keywords:** acute pulmonary embolism, computer tomographic pulmonary angiography, intramuscular adipose tissue, machine learning, radiomics, skeletal musculature

## Abstract

**Background:**

Acute pulmonary embolism (APE) is a potentially life‐threatening disorder, emphasizing the importance of accurate risk stratification and survival prognosis. The exploration of imaging biomarkers that can reflect patient survival holds the potential to further enhance the stratification of APE patients, enabling personalized treatment and early intervention. Therefore, in this study, we develop computed tomography pulmonary angiography (CTPA) radiomic signatures for the prognosis of 7‐ and 30‐day all‐cause mortality in patients with APE.

**Methods:**

Diagnostic CTPA images from 829 patients with APE were collected. Two hundred thirty‐four features from each skeletal muscle (SM), intramuscular adipose tissue (IMAT) and both tissues combined (SM + IMAT) were calculated at the level of thoracic vertebra 12. Radiomic signatures were derived using 10 times repeated three‐fold cross‐validation on the training data for SM, IMAT and SM + IMAT for predicting 7‐ and 30‐day mortality independently. The performance of the radiomic signatures was then evaluated on held‐out test data and compared with the simplified pulmonary embolism severity index (sPESI) score, a well‐established biomarker for risk stratification in APE. Predictive accuracy was assessed by the area under the receiver operating characteristic curve (AUC) with a 95% confidence interval (CI), sensitivity and specificity.

**Results:**

The radiomic signatures based on IMAT and a combination of SM and IMAT (SM + IMAT) achieved moderate performance for the prediction of 30‐day mortality on test data (IMAT: AUC = 0.68, 95% CI [0.57–0.78], sensitivity = 0.57, specificity = 0.73; SM + IMAT: AUC = 0.70, 95% CI [0.60–0.79], sensitivity = 0.74, specificity = 0.54). Radiomic signatures developed for predicting 7‐day all‐cause mortality showed overall low performance. The clinical signature, that is, sPESI, achieved slightly better performance in terms of AUC on test data compared with the radiomic signatures for the prediction of both 7‐ and 30‐day mortality on the test data (7 days: AUC = 0.73, 95% CI [0.67–0.79], sensitivity = 0.92, specificity = 0.16; 30 days: AUC = 0.74, 95% CI [0.66–0.82], sensitivity = 0.97, specificity = 0.16).

**Conclusions:**

We developed and tested radiomic signatures for predicting 7‐ and 30‐day all‐cause mortality in APE using a multicentric retrospective dataset. The present multicentre work shows that radiomics parameters extracted from SM and IMAT can predict 30‐day all‐cause mortality in patients with APE.

## Introduction

Acute pulmonary embolism (APE) is a potentially life‐threatening disorder.^1^, ^2^ Therefore, accurate methods for risk stratification and survival prognosis for patients with APE may allow for early intervention and personalized treatment management. Few clinical and molecular biomarkers have been identified for prognosticating short‐term mortality. Among clinical biomarkers, simplified pulmonary embolism severity index (sPESI) score^3^ and Hestia criteria^4^ are significantly associated with mortality in APE patients (Hestia criteria: pooled odds ratio [OR] = 6.120, 95% confidence interval [CI] [2.90–12.89]; sPESI score: pooled OR = 12.74, 95% CI [3.98–40.77]).^5^ Among molecular biomarkers, troponin and brain natriuretic peptide (BNP) are the most commonly utilized molecular biomarkers for short‐term risk stratification in APE.^6^ Imaging biomarkers reflecting patient survival may help to further improve patient stratification.

Computed tomography pulmonary angiography (CTPA) is the gold standard for the diagnosis of APE and predicts clinical outcomes in APE. A high ratio of right ventricle (RV) to left ventricle (LV) diameter on CTPA is strongly associated with mortality in APE (pooled OR = 2.5, 95% CI [1.80–3.50], *P* < 0.001).^7^ Furthermore, the presence of septal straightening/bowing is also associated with all‐cause mortality (pooled OR = 1.7, 95% CI [1.2–2.4], *P* = 0.002).^7^

According to the literature, reduced mass of the skeletal musculature plays an important role in the diagnosis and prognosis of several acute diseases. For instance, it can be used as a surrogate parameter for sarcopenia,^8^ and it is a strong predictor of 30‐day mortality in patients undergoing emergency laparotomy (OR = 10.1, 95% CI [5.6–17.7], *P* < 0.001).^9^ Furthermore, reduced muscle mass and/or density is associated with in‐hospital mortality in patients with COVID‐19 infection (hazard ratio [HR] = 5.84, 95% CI [1.07–31.83], *P* = 0.04).^10^ Also in APE, sarcopenia is associated with 30‐day mortality, although the identified effect is low (HR = 1.06, 95% CI [1.03–1.09], *P* < 0.001).^11^ Thus, additional investigation is required to establish the correlation between skeletal musculature and APE.

Currently, modern imaging analysis, such as radiomics, is used to identify quantitative imaging biomarkers by employing statistical and machine learning algorithms. The use of radiomics has shown remarkable potential for patient survival prediction, especially in the context of oncology.^12^, ^13^ In the context of APE, studies have shown that radiomic features derived from thrombotic clots in CTPA images differed significantly between survivors and non‐survivors.^14^ However, to the best of our knowledge, the association between skeletal musculature and patient survival in APE is not yet established with radiomics.

Therefore, in this work, we investigate the prognostic role of radiomics‐based features of the skeletal muscle (SM) and intramuscular adipose tissue (IMAT) for predicting 30‐day all‐cause mortality in patients with APE using multicentric CTPA imaging data.

## Material and methods

### Patient data

Ethics approval for multicentric retrospective analysis was obtained from the Ethics Committee (Medical Faculty, Otto‐von‐Guericke‐University Magdeburg, Number 145‐21). In this retrospective study, imaging and clinical data of 981 APE patients were included from three different sites in Germany. Of these, 556 patients (56.7%) were treated at the University Hospital Magdeburg (Centre 1) between 2015 and 2021, 226 patients (23.0%) were treated at the University Hospital Leipzig (Centre 2) between 2012 and 2017, and 199 patients (20.3%) were treated at the University Hospital Halle (Centre 3) between 2005 and 2010. The inclusion criteria for the present study were (i) age ≥ 18 years and (ii) evidence of APE on CTPA. Patients with chronic pulmonary embolism (PE) and significant artefacts from CTPA images were excluded from the analysis. Also, patients with brain infarction, neuromuscular disorders and traumatic injuries of the trunk and/or spine were excluded. Consequently, 152 (15.49%) patients who did not meet the inclusion criteria were excluded from the analysis, with 62 (6.32%) patients being excluded specifically due to CTPA image artefacts. This led to the inclusion of 829 patients in total: Centre 1 (450 patients, 54.3%), Centre 2 (210 patients, 25.3%) and Centre 3 (169 patients, 20.4%). Subsequently, the data of the included patients were divided into training and test datasets using a 70/30 ratio, stratified by outcome, resulting in 580 patients for training and 249 patients for testing. The characteristics of the patients are summarized in *Table* *1*.

**Table 1:** Patient characteristics for the training and test data

Variable	Training data (580)		Test data (249)		*P*‐value
	Median	Range	Median	Range	
Age (years)	65	15–97	65	18–100	0.98
	**Number**	**%**	**Number**	**%**	
Gender					
Male	314	54.14	130	52.21	0.66
Female	266	46.86	119	47.79	
sPESI					
0	104	17.93	37	14.86	0.54
1	171	29.48	83	33.33	
2	169	29.14	71	28.51	
3	95	16.38	40	16.06	
4	37	6.38	13	5.22	
5	3	0.52	4	1.61	
6	1	0.17	1	0.40	
30‐day mortality					
Dead	86	14.83	35	14.06	0.85
Alive	494	85.17	214	85.94	
7‐day mortality					
Dead	47	8.10	23	9.24	0.70
Alive	533	91.90	226	90.76	

Abbreviation: sPESI, simplified pulmonary embolism severity index.

The primary endpoint of this study was 7‐ and 30‐day all‐cause mortality. The endpoint for analysis was defined as a binary outcome, where a class label of 1 indicated patients who experienced an observed event (death) within 7 and 30 days of diagnosis, while a class label of 0 denoted patients who were alive after 7 and 30 days. The following baseline clinical parameters were included in the study: gender, age and sPESI. The sPESI score includes six equally weighted variables as follows: age > 80 years (1 point), presence of cancer (1 point), chronic heart failure or chronic pulmonary disease (1 point), systolic blood pressure < 100 mmHg (1 point), and arterial oxyhaemoglobin saturation < 90% (1 point).

### Study design

**Figure 1:**
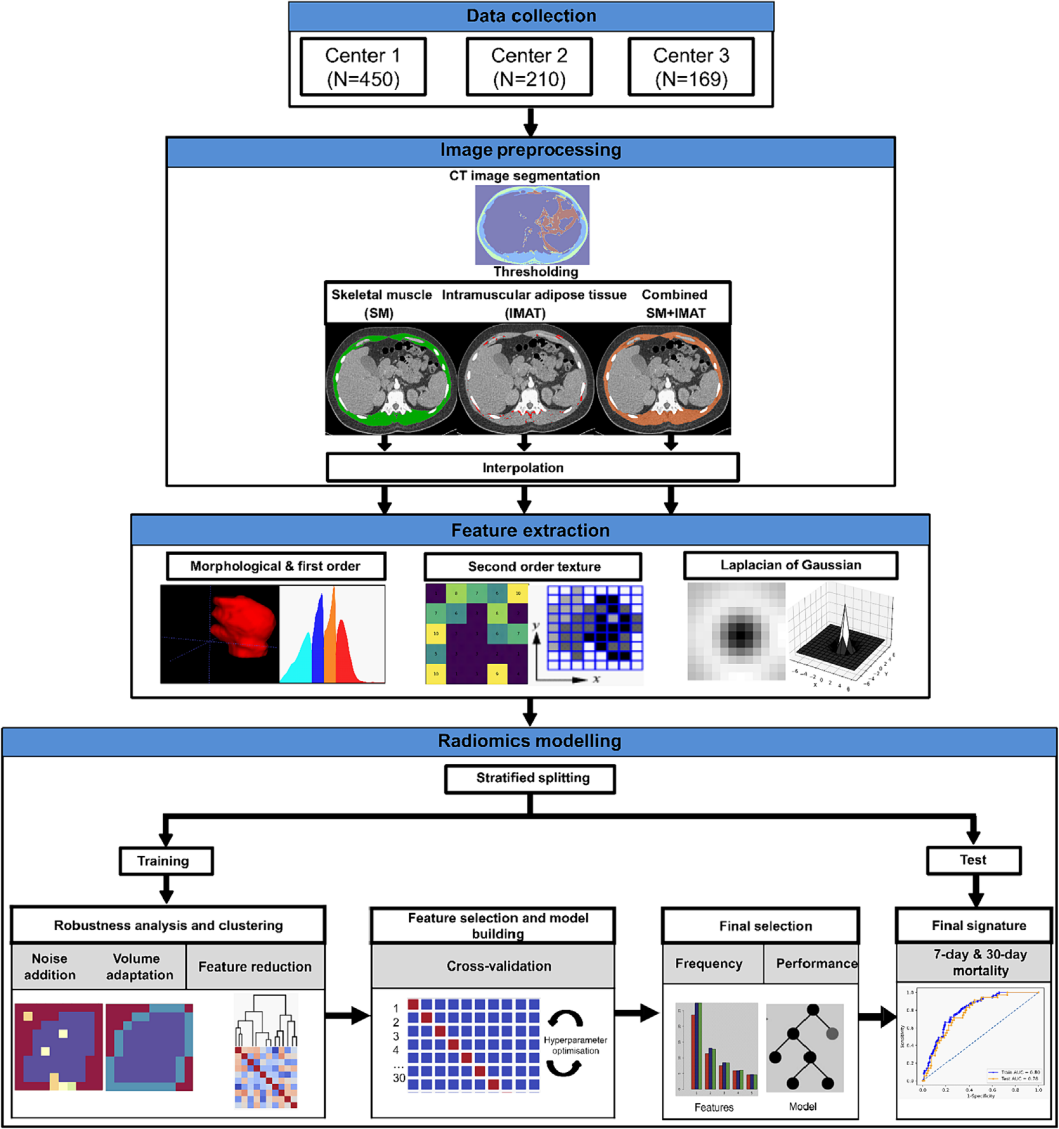
Overview of the study workflow. Computed tomography pulmonary angiography (CTPA) data were collected from three centres (University Hospital Magdeburg [Centre 1, *N* = 450], University Hospital Leipzig [Centre 2, *N* = 210] and University Hospital Halle [Centre 3, *N* = 169]) and randomly assigned to training and test datasets using a stratified 70/30 split. Skeletal muscle (SM) and intramuscular adipose tissue (IMAT) were segmented at the level of thoracic vertebra 12 (Th12) on a single axial slice of diagnostic CTPA. All CTPA images were then preprocessed by applying thresholding and interpolation, followed by radiomic feature extraction. Features were extracted from the region of interest (ROI) defined by SM, IMAT and both tissues combined (SM + IMAT). Radiomic signatures were established for each ROI after removing non‐robust and redundant features using automated machine learning on the training data. The final signature was then used to train a classifier and validated on test data for predicting both 7‐ and 30‐day mortality.

In this study, we developed and validated radiomic signatures for the prognosis of 7‐ and 30‐day all‐cause mortality in patients with APE using diagnostic CTPA. *Figure* *1* summarizes the design of our study.

In short, imaging features were computed from a thoracic muscle area segmented at the level of thoracic vertebra 12 (Th12) on a single axial slice of a diagnostic CTPA from SM, IMAT and both tissues combined (SM + IMAT). Imaging features included first‐order features (local, statistical and intensity histograms and intensity volume histograms), second‐order texture (SOT) features and Laplacian of Gaussian (LoG) transformed intensity features. Non‐robust features were removed through image perturbation, and then redundant features were eliminated through clustering. Remaining features were used to create radiomic models based on 10 times repeated three‐fold cross‐validation (CV) of the training data. Radiomic models were developed on the training data using three different machine learning models of varying complexity: logistic regression (GLM_logistic), gradient‐boosted linear model (XGB_lm) and random forest (RF). A radiomic signature was then identified based on the occurrence of features within the models in the CV experiment. This signature was then subjected to testing, and its performance was compared with the baseline clinical signature, that is, the sPESI score, for predicting 30‐day all‐cause mortality in patients with APE. The performance of the signature was assessed on the separate test data using the area under the curve (AUC), sensitivity and specificity metrics.

The following sections provide a breakdown of the steps involved in image preprocessing, the extraction of radiomic features and the subsequent modelling process.

### Image acquisition, image preprocessing and feature extraction

The clinical database of each participating centre was retrospectively screened for patients with APE. CTPA in this retrospective cohort was performed with intravenous administration of an iodine‐based contrast medium. *Table* *S1* summarizes CTPA image acquisition and reconstruction parameters for each centre. SM and IMAT were segmented by an experienced radiologist (M.H.) on a single axial slice of computed tomography (CT) at the level of Th12 with the freely available ImageJ software Version 1.53 (National Institutes of Health, Bethesda, MD, USA). The segmentation process using ImageJ involved applying specific threshold values to distinguish different tissue types. SM was segmented using a threshold range of −29 Hounsfield units (HU) to 150 HU. Additionally, the fat fraction within the skeletal musculature area (IMAT) was segmented using a threshold range of −190 to −30 HU. This method enabled the precise delineation of SM and adipose tissue compartments within the CT. The segmentation quality for each patient was then checked by an experienced radiologist (J.B.) for the entire data.

Image preprocessing followed by feature extraction was carried out using the open‐source Medical Image Radiomics Processor (MIRP) Python toolkit Version 1.1.^15^ CTPA image voxels were resampled to an isotropic voxel size of 1.0 × 1.0 mm using cubic interpolation to harmonize the voxel spacing between the datasets. Based on segmentations obtained from ImageJ software, features were extracted individually from SM (−29 to +150 HU), IMAT (−190 to −30 HU) and both tissues combined (−190 to +150 HU). An example of each segmented region of interest (ROI) used for feature extraction is shown in *Figure* *S1*. A set of LoG filters with five different kernel widths (1, 2, 3, 4 and 5 mm) was applied individually to the baseline segmented CTPA images. The five response maps were averaged into a single image. After image preprocessing, imaging features were computed. A set of 25 morphological and 57 first‐order features (MFO features), as well as 95 SOT features, were calculated from the single 2D CTPA slice per patient. Finally, the same 57 first‐order features were extracted from the LoG transformed images (LoG features). Consequently, 234 radiomic features were extracted from each SM, IMAT and SM + IMAT segmentation at the Th12 level from a 2D CTPA slice per patient. SOT features were extracted from the 2D ROI based on the grey level co‐occurrence matrix (GLCM), grey level run length matrix (GLRLM), grey level size zone matrix (GLSZM), grey level distance zone matrix (GLDZM), neighbourhood grey tone dependence matrix (NGTDM) and neighbouring grey level dependence matrix (NGLDM). Image preprocessing and feature extraction in MIRP were implemented according to the Image Biomarker Standardization Initiative (IBSI) reference standards.^16^, ^17^ Image processing parameters used for feature extraction are summarized in *Table* *S2*.

In order to obtain reproducible results, imaging features have to be stable under image variations, for example, those caused by differing acquisition parameters or positioning variations.^15^ We evaluated feature robustness by applying the following image augmentations based on the training data: adding Gaussian noise (mean 0, standard deviation as present in the image) and random volume changes of the ROI (−10%, −5%, 0%, 5% and 10%). The resulting five perturbed images for each original CT image were then used to extract features for robustness analysis. The intra‐class correlation coefficient (ICC) was calculated for perturbed features with a 95% CI, quantifying the similarity of feature values under different perturbations for every feature. Features with the lower boundary of the 95% CI of the ICC below 0.8 were removed from the feature set computed on baseline segmented CTPA images.

After robustness analysis, redundant features were identified and removed by hierarchical clustering. The Spearman correlation coefficient was used as a similarity metric, with average linkage as a criterion for merging two clusters. A mutual Spearman correlation of 0.8 was defined for placing features in the same cluster. The feature with the highest mutual information with the endpoint was selected as the representative for each cluster.

Robustness analysis followed by clustering reduced features to 46 (SM), 41 (IMAT) and 45 (SM + IMAT) features. Based on these reduced feature sets, radiomic signatures were developed and tested for 7‐ and 30‐day mortality in APE. The details regarding radiomics modelling are presented in detail in the following section.

### Radiomics modelling

In our analysis, we evaluated the prognostic performance of CTPA radiomic signatures for the prediction of 7‐ and 30‐day all‐cause mortality in patients with APE. Briefly, to create radiomic signatures for the individual ROI, that is, SM, IMAT and both tissues combined (SM + IMAT), a workflow containing four major processing steps was applied after feature clustering using the open‐source end‐to‐end statistical learning software package Familiar (1.4.1)^18^: (i) feature preprocessing, (ii) feature selection, (iii) model building with internal validation and (iv) testing. Steps (i)–(iii) were first performed using 10 repetitions of three‐fold stratified CV, nested in the training dataset, to identify an optimal signature. After identifying the final signature and best performing learner using the training dataset, a final model was developed on the entire training data and validated on the held‐out test data.

The following procedure was performed for each of the 30 CV runs: (i) Features were transformed using the Yeo–Johnson transformation to align their distribution to a normal distribution. Afterwards, features were z‐transformed to mean zero and standard deviation one. Both transformations were performed on the internal training part and applied unchanged to the features of the internal validation part. (ii) Three supervised feature‐selection algorithms were considered: minimal redundancy maximum relevance (MRMR),^19^ mutual information maximization (MIM)^20^ and univariate regression.^21^ To avoid overfitting, only the five most relevant features were selected for each CV fold. (iii) The selected features were used by three different classifiers: logistic regression (GLM_logistic),^21^ a gradient‐boosted linear model (XGB_lm)^22^ and RF^23^ for the detection of 7‐ and 30‐day all‐cause mortality separately.

The hyperparameters of the classifiers were tuned automatically using a sequential model‐based optimization algorithm based on bootstrap sampling of the training data.^24^ Each classifier was built on the internal training folds, and its performance was assessed on the internal validation fold. For every feature‐selection method, average model performance was assessed by the median AUC. After CV, for each of the feature‐selection methods, features were ranked according to their occurrence score across the 30 CV runs. Features that showed occurrence scores above 50% in each feature‐selection method and showed a repeated occurrence in at least two out of three feature‐selection methods were selected. If a subset of these features showed a Spearman correlation |*ρ*| > 0.5 with each other on the entire training data, only the feature with the highest occurrence was considered. A detailed example of the feature‐selection scheme for the prediction of 30‐day mortality using features extracted from SM + IMAT is shown in *Table* *S3* and *Figure* *S2*.

The resulting radiomic signature was then used to build a classification model using the learner that showed the overall highest performance in terms of AUC in CV folds. The final radiomics model was built on the entire training data, and (iv) the trained model was applied to the held‐out test data.

Finally, the classification model was trained using the best performing learners and applied to the held‐out test data using the final radiomic signature.

### Statistical analysis

Categorical variables of the clinical data were compared between the training and test data by the *χ* ^2^ test, whereas continuous variables were compared using the Mann–Whitney *U* test.

Associations between the final model predictions and the endpoint were evaluated using AUC, sensitivity and specificity metrics. The estimated value and the 95% CI of the AUC were computed using the bias‐corrected bootstrap CI method^25^ and compared using the DeLong test. For creating a confusion matrix based on the final signature for predicting 30‐day mortality in patients with APE, an optimal cut‐off for radiomic signature was selected on the training data that maximize Youden's index (J) and transferred to the held‐out test data. Calibration for the model predictions was assessed via the Hosmer–Lemeshow goodness‐of‐fit test (HL test).^26^ Correlations between features were assessed by the Spearman correlation coefficient (*ρ*). All tests were two‐sided, with a significance level of 0.05. The importance of individual features in the final signature was assessed by univariate fitting of a logistic regression model and computing the Wald test *P*‐values. Analyses were performed in R Version 4.2.3.

## Results

Patient characteristics of the training and test data are summarized and compared in *Table* *1*. Overall, 829 patients (444 male patients [53.56%] and 385 female patients [46.44%]) with a median age of 65 (range [18–100]) years and sufficient clinical and imaging data were used for training and testing of radiomics models. Overall, 70 patients (8.44%) and 121 patients (14.60%) of the investigated 829 patients died within the 7‐ and 30‐day periods, respectively. There was no significant difference between patient characteristics in training and test data.

In internal CV, the overall highest classifier performance for the prediction of 7‐ and 30‐day mortality with radiomics modelling was observed for the XGB_lm model with all feature‐selection methods compared with the GLM_logistic and RF models. The performance of the considered models for each feature‐selection method in the internal CV for both 7‐ and 30‐day mortality is shown in *Table* *S4* and *Figure* *S3*.

*Table* *2* shows the results for radiomic and clinical signatures. Given the higher performance of the XGB_lm classifier in internal CV, it was employed to construct final radiomics models using the selected signature to build a prognostic model on the entire training data and applied to the held‐out test data. On test data, the radiomics model constructed solely from SM tissue showed overall lower performance for predicting both 7‐day mortality (training: AUC = 0.71, 95% CI [0.64–0.77]; test: AUC = 0.56, 95% CI [0.43–0.69]) and 30‐day mortality (training: AUC = 0.73, 95% CI [0.67–0.78]; test: AUC = 0.64, 95% CI [0.53–0.74]). Similarly, a radiomics model based solely on a signature constructed from IMAT showed lower predictive performance for 7‐day mortality on test data (training: AUC = 0.70, 95% CI [0.63–0.77]; test: AUC = 0.62, 95% CI [0.50–0.74]) but relatively improved performance for 30‐day mortality (training: AUC = 0.73, 95% CI [0.67–0.79]; test: AUC = 0.68, 95% CI [0.57–0.78]). Finally, the radiomics model incorporating features from both SM and IMAT (SM + IMAT) also showed lower predictive performance for 7‐day mortality on test data (training: AUC = 0.74, 95% CI [0.68–0.80]; test: AUC = 0.57, 95% CI [0.46–0.67]), while demonstrating the highest performance for predicting 30‐day mortality (training: AUC = 0.77, 95% CI [0.68–0.80]; test: AUC = 0.70, 95% CI [0.60–0.79]). At a cut‐off value of 0.33, the SM + IMAT model for predicting 30‐day mortality was able to achieve sensitivity and specificity of 0.74 and 0.54, respectively, on held‐out test data.

**Table 2:** Area under the curve (AUC), sensitivity, specificity and cut‐off values for 7‐ and 30‐day all‐cause mortality prediction in acute pulmonary embolism patients based on clinical and radiomic signatures

Signature	Features	Mortality	Final training AUC	Final test AUC	Sensitivity/specificity train	Sensitivity/specificity test	Cut‐off
Clinical only	sPESI	7 days	0.74 (0.66–0.82)	0.73 (0.67–0.79)	0.95/0.19	0.92/0.16	1.00
		30 days	0.72 (0.67–0.77)	0.74 (0.66–0.82)	0.99/0.21	0.97/0.16	1.00
Radiomics SM	cm_sum_avg_d1_2d_s_mrg_fbn morph_pca_elongation	7 days	0.71 (0.64–0.77)	0.56 (0.43–0.69)	0.77/0.58	0.52/0.58	0.46
	Ivh_i50 morph_pca_elongation	30 days	0.73 (0.67–0.78)	0.64 (0.53–0.74)	0.73/0.62	0.63/0.64	0.14
Radiomics IMAT	morph_comp_1 stat_qcod	7 days	0.70 (0.63–0.77)	0.62 (0.50–0.74)	0.72/0.62	0.74/0.54	0.08
	stat_qcod ngl_glnu_d1_a0_0_2d_fbn_n24 morph_pca_elongation	30 days	0.73 (0.67–0.79)	0.68 (0.57–0.78)	0.66/0.75	0.57/0.73	0.22
Radiomics SM + IMAT	stat_rms szm_sze_2d_fbn_n24	7 days	0.74 (0.68–0.80)	0.57 (0.46–0.67)	0.93/0.44	0.65/0.42	0.49
	stat_skew szm_sze_2d_fbn_n24 morph_pca_elongation	30 days	0.77 (0.72–0.81)	0.70 (0.60–0.79)	0.86/0.58	0.74/0.54	0.33

*Note*: Radiomic signatures were built from features extracted from skeletal muscle (SM), intramuscular adipose tissue (IMAT) and a combination of both (SM + IMAT). The values in parentheses represent the 95% confidence interval for AUC values. Abbreviation: sPESI, simplified pulmonary embolism severity index.

**Figure 2:**
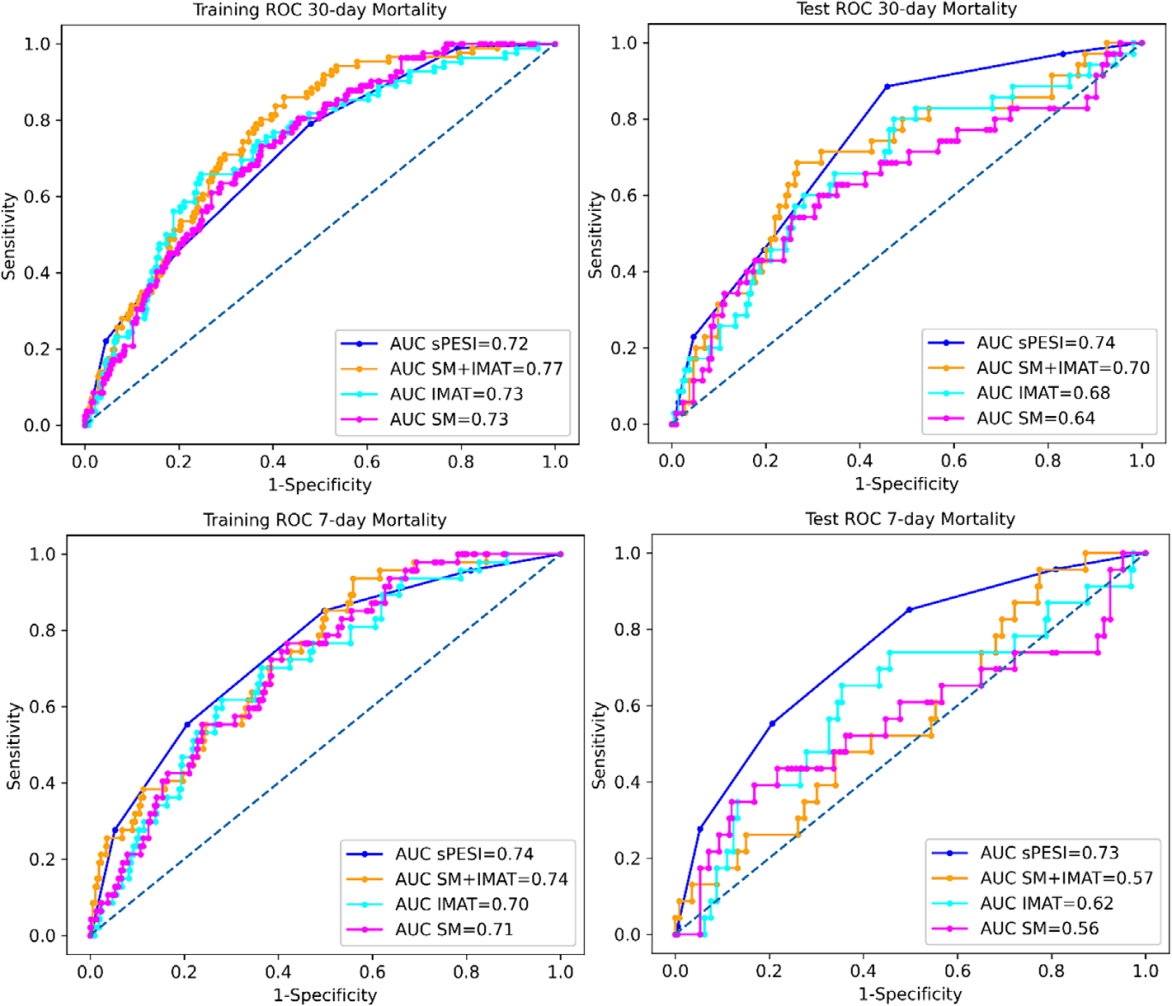
Receiver operating characteristic (ROC) curves with corresponding area under the curve (AUC) values on training and test data for prediction of 30‐ and 7‐day all‐cause mortality in patients with acute pulmonary embolism (APE) resulting from clinical signatures (sPESI) and radiomic signatures developed from skeletal muscle (SM), intramuscular adipose tissue (IMAT) and both tissues combined (SM + IMAT).

The clinical model containing only sPESI score was able to achieve better performance on test data in terms of AUC compared with radiomics models for predicting both 7‐day mortality (training: AUC = 0.74, 95% CI [0.66–0.82]; test: AUC = 0.73, 95% CI [0.67–0.79]) and 30‐day mortality (training: AUC = 0.72, 95% CI [0.67–0.77]; test: AUC = 0.74, 95% CI [0.66–0.82]). At the cut‐off value of sPESI score 1, the clinical model achieved lower specificity (0.16 for both 7‐ and 30‐day mortality) but significantly higher sensitivity (0.92 for 7‐day mortality and 0.97 for 30‐day mortality) on test data. *Figure* *2* shows the comparison between the receiver operating characteristic (ROC) curves of the developed models: the clinical model based on sPESI score and the radiomics models utilizing SM, IMAT and SM + IMAT features, aimed at predicting 30‐ and 7‐day mortality. The ROC‐AUC comparison using the DeLong test revealed no significant difference between models for predicting 30‐day mortality in training and test data (*P*‐value > 0.05). For predicting 7‐day mortality, the DeLong test revealed a significant difference between clinical (sPESI) and SM + IMAT model predictions (*P*‐value = 0.03) and clinical (sPESI) and SM model predictions (*P*‐value = 0.05). The DeLong test results are shown in *Table* *S5*.

**Figure 3:**
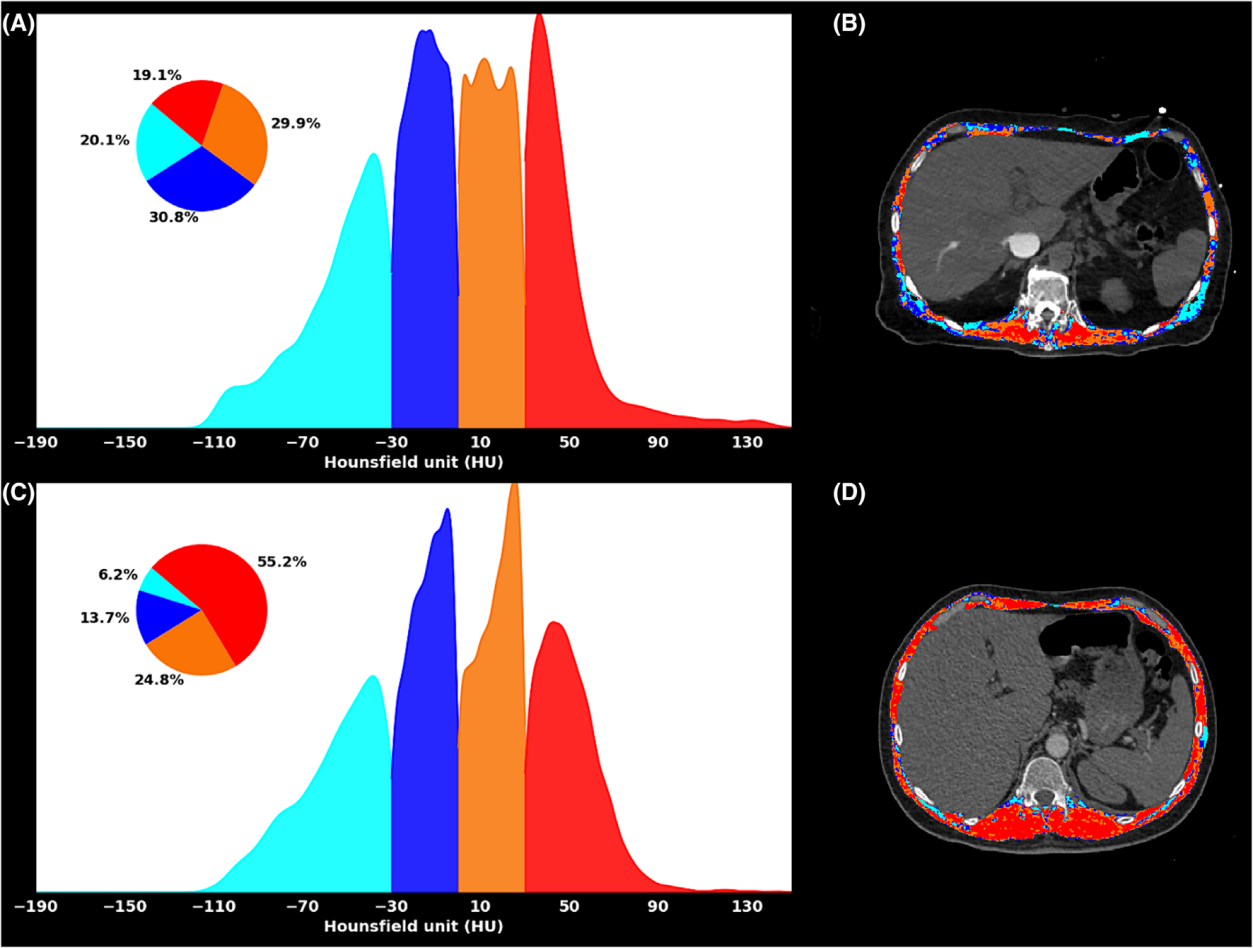
Representative computed tomography pulmonary angiography (CTPA) images from two APE patients, that is, (A, B) Patient 1 with survival ≤ 30 days and (C, D) Patient 2 with survival ≥ 30 days. Patient 1 (outcome: death; age = 78 years) showed visible intramuscular fat accumulation (IMAT) (light blue) within the skeletal muscle (SM). On the contrary, Patient 2 (outcome: alive; age = 53 years) showed a more homogenous region of interest (ROI) with a higher percentage of normal muscles, causing intensities to be negatively skewed compared with intensity distribution in Patient 1. Further, Patient 2 shows a normal range of muscle radiation HU values, resulting in a fragmented or scattered distribution of grey levels with small zones compared with Patient 2. Further, Patient 1 has a more elongated ROI compared with Patient 2.

The best performing radiomics model was based on three features extracted from SM + IMAT. The selected CT features, namely, stat_skew (IBSI: C317), morph_pca_elongation (IBSI: Q3CK) and szm_sze_2d_fbn_n24 (IBSI: 5QRC), were selected for final signature and examined further for their association with the endpoint. stat_skew represents the skewness of the discretized histogram (24 bins) derived from the baseline 2D CTPA images. High values of stat_skew indicate a positively skewed intensity distribution within the ROI. In patients with APE (survival ≤ 30 days), the stat_skew feature revealed a higher concentration of intramuscular fat (20.1) with only 19.1% of the total muscle falling into the normal attenuation range (30–150 HU), resulting in a positively skewed intensity distribution (*Figure* *3* *A,B*). Conversely, patients with survival ≥ 30 days exhibited negative skewness due to a lower concentration of intramuscular fat (6.2%) but a higher concentration (55.2%) of healthy muscles (*Figure* *3* *C,D*). morph_pca_elongation represents the extent to which an ROI is elongated, with smaller values indicating greater elongation, while a value of 1 indicates a perfectly circular ROI. APE patients with survival ≤ 30 days showed lower values of morph_pca_elongation, indicating greater elongation of the abdominal region, potentially related to muscle loss and weakness (*Figure* *3* *B*). szm_sze_2d_fbn_n24 is a GLSZM feature that emphasizes small zones of similar grey level values compared with larger zones computed on a 2D image with a discretized histogram (24 bins). APE patients with survival ≤ 30 days exhibited more variation in tissue make‐up, with many small regions of similar HU values, compared with less acute patients. This feature is another indicator of intramuscular fat accumulation, causing the formation of small zones with similar grey levels. *Figure* *3* *A–D* visually illustrates these findings in two patients' groups. The corresponding boxplots of selected features are shown in *Figure* *S4*.

All the selected features showed a significant contribution in both training and testing (*P* < 0.01). The XGB_lm model parameters for the best performing signatures from SM + IMAT are presented in *Table* *S6*. *Figure* *S5* presents the calibration plots for best performing radiomics (SM + IMAT) and clinical (sPESI) signatures on training and test data.

## Discussion

In this study, we developed radiomic signatures incorporating CTPA features for the prediction of 7‐ and 30‐day all‐cause mortality in patients with APE using features from SM, IMAT and both tissues combined (SM + IMAT). The predictive performance of clinical and radiomic signatures was tested independently. Radiomic signature comprising three explainable CTPA features extracted from ROI defined by IMAT and SM + IMAT showed acceptable performance for the prediction of 30‐day all‐cause mortality on test data (IMAT: AUC = 0.68, 95% CI [0.57–0.78], sensitivity = 0.57, specificity = 0.73; SM + IMAT: AUC = 0.70, 95% CI [0.60–0.79], sensitivity = 0.74, specificity = 0.54). Radiomic signatures constructed solely from SM tissue exhibited lower overall performance. None of the radiomics models demonstrated acceptable performance for predicting 7‐day all‐cause mortality in APE.

Risk stratification in APE is useful for deciding the optimal treatment strategy. Patients with a high risk of mortality require immediate hospitalization and potential primary or rescue therapy, while those at low risk without ventricular dysfunction can be treated as outpatients.^27^ Several prediction models combining clinical variables, molecular biomarkers and medical imaging have been developed to predict short‐term mortality and basically identify risk groups in APE. Among clinical biomarkers, the sPESI score, which combines six clinical variables, is extensively used for risk stratification in APE. According to a 2012 meta‐analysis, sPESI was able to predict all‐cause mortality with an AUC of 0.79, a pooled sensitivity of 0.92 and a pooled specificity of 0.38.^28^ In our training and test data, the predictive performance of the sPESI score is somewhat lower for predicting 30‐ and 7‐day all‐cause mortality (7 days: train/test AUC = 0.74/0.73, train/test sensitivity = 0.98/0.96, train/test specificity = 0.19/0.16; 30 days: train/test AUC = 0.72/0.74, train/test sensitivity = 0.99/0.97, train/test specificity = 0.21/0.16). The lower sensitivity in our cohort can be attributed to a data imbalance, with a significantly lower number of patients with survival of ≤7 and 30 days. Further, score‐based stratification relies heavily on demographic and co‐morbid conditions. Therefore, clinical scores alone are insufficient to guide personalized treatment. Among molecular biomarkers, the prognostic value of BNP and its N‐terminal portion (NT‐proBNP) have been evaluated in several studies.^29^ Exemplary studies by Söhne et al.^30^ and Lankeit et al.^31^ showed that BNP (AUC = 0.63) and NT‐proBNP (AUC = 0.72) provide prognostic value for risk stratification in APE. More recently, echocardiographic markers such as subcostal echocardiographic assessment of tricuspid annular kick (SEATAK) have been reported to be associated with mortality in APE (AUC = 0.72).^32^ Among imaging markers, a large meta‐analysis found that increased RV to LV diameter (RV:LV) ratio measured on axial CTPA images is the most powerful independent predictor of all‐cause mortality in APE (pooled OR = 2.5, 95% CI [1.8–3.5], *P* < 0.001).^7^ More recently, it has been shown that deep learning‐based automatic ventricle detection and volume segmentation followed by RV:LV ratio calculation achieved an AUC of 0.77 for predicting mortality in APE.^33^ Despite the consistent molecular and imaging markers associated with short‐term mortality in APE, they appear clinically insufficient to guide the initiation of personalized therapy in APE, and further analysis is required in this regard. In our radiomics analysis based on CTPA images, our radiomic signature showed comparable results to previous studies (radiomics AUC = 0.70). This indicates that radiomics have the potential to increase the prognostic value of short‐term mortality in APE.

Several studies have explored the muscle quality reflected by imaging features for prognosis in cancer.^34^ For instance, sarcopenia, that is, the decrease in muscle mass and strength, was found to be significantly associated with all‐cause mortality in hepatocellular carcinoma (HCC) patients (adjusted HR = 1.95, 95% CI [1.60–2.37]).^35^ The muscle volume for this association is often estimated on the transverse CT image at the third lumbar vertebra, with an intensity window for recognizing SMs varying between −30 and 150.^36^ However, radiomics‐based analysis of body composition and its association with patient prognosis is rarely performed in the literature. Only recently, a study by Saalfeld et al.^37^ showed that radiomic features extracted from SM and adipose tissues can predict 1‐year survival in HCC patients (AUC = 0.76, 95% CI [0.64–0.88]). Therefore, in the context of APE, we hypothesized that conducting an in‐depth examination of SM and adipose tissue quality with radiomics may also yield new, significant parameters with predictive potential, and our results confirm our hypothesis. To the best of our knowledge, this is the first investigation of the prognostic role of radiomics‐based values of the SM and IMAT in APE. Our SM + IMAT radiomics model exhibited lower sensitivity but improved specificity to the specific clinical score sPESI. This underscores the prognostic value provided by radiomics analysis in APE. Further, we successfully provide a visual interpretation of our radiomic signature. Specifically, two of the features stat_skew and szm_sze_2d_fbn_n24 provide a quantitative measurement of the intramuscular fat contents (IMAT) of SM, high levels of which are implicated in a number of diseases and dysfunctions.^38^ Further, the radiomics model of IMAT also showed better performance on test data in terms of AUC = 0.68 (95% CI [0.57–0.78]) for predicting 30‐day all‐cause mortality. It appears reasonable to hypothesize that individuals exhibiting elevated intramuscular fat levels may generally have poorer health status, consequently increasing their susceptibility to mortality following an episode of APE. However, the radiomics model based solely on features extracted from SM showed lower overall performance. This underscores the significance of analysing both SM and IMAT combined. We also noted that the morphological elongation feature (morph_pca_elongation), indicative of greater elongation of the abdominal region possibly due to muscle loss and weakness in high‐risk patients after APE, was selected in most models. Therefore, it is crucial to investigate both morphological and texture features to develop improved prognostic models for mortality in APE. However, understanding the relationship between these features and biological variations is challenging based solely on the data presented in this study. Further investigation into the underlying biological variation of radiomic features within muscle tissue is warranted. For future research, magnetic resonance imaging (MRI) may also be considered for imaging patients with APE, given its superior soft‐tissue contrast, particularly for fatty tissues.

Associations between muscle quality reflected by radiomic parameters and survival in patients with APE are multicausal. It is well known that myocardial dysfunction, especially RV dysfunction, plays a key clinical role and predicts relevant outcomes in patients with APE.^7^ We hypothesize that the status of the skeletal musculature may be associated with the status of the myocardium. In fact, SMs produce and release several cytokines (myokines) with protective effects on the cardiovascular system.^39^, ^40^, ^41^, ^42^, ^43^ The important cardioprotective myokines are irisin, musclin, myonectin, follistatin‐like 1 factor and leukaemia inhibitory factor. For instance, irisin significantly reduces infarct size and post‐myocardial cardiac fibrosis.^43^ Also, irisin administration significantly increases angiogenesis in the infarct border zone and decreases cardiomyocyte apoptosis.^43^ Intravenous administration of irisin protects against ischaemia/reperfusion‐induced injury of the lung.^39^ Another myokine, musclin, has a myocardial protective effect against injury from ischaemia and pressure overload.^40^, ^41^ A similar effect is also observed for myonectin.^42^ Furthermore, myonectin reduces cardiomyocyte apoptosis.^42^ Follistatin‐like 1 factor suppresses apoptosis of cardiomycites.^43^ Finally, leukaemia inhibitory factor improves cardiac recovery and enhances the proliferation of cardiomyocytes following myocardial infarction.^43^

Presumably, alteration and low quality of the skeletal musculature may result in low secretion of myokines. A low level of myokines may be associated with cardiovascular events. In fact, some studies confirm this assumption. So far, brain‐derived neurotropic factor (BDNF), a member of the neurotrophic factor family, is a myokine that influences the central nervous system.^44^ This myokine also influences the cardiovascular system. Low plasma BDNF levels are associated with coronary events and mortality in patients with angina pectoris.^44^ Radiomics parameters may reflect deep changes and metabolic activity of the skeletal musculature. Previously, Bhullar et al. showed a very heterogenous distribution of lipids within the skeletal musculature.^45^ Radiomics parameters may reflect intramuscular deep changes and heterogeneity of lipid distribution. Also, radiomics values may be associated with the metabolic activity of the skeletal musculature.

Importantly, radiomic features of the skeletal musculature were more sensitive for prediction of an unfavourable prognosis in patients with APE in comparison with ‘conventional values’ like SM area and density, which were used in our previous investigation.^46^ In fact, our present analysis showed that values for the radiomics‐based analysis are superior to those reported for the conventional analysis. Our study represents the first attempt to apply radiomics techniques to predict 30‐day mortality in patients with PE. While the association between intramuscular fat and mortality has been reported in the literature, our study extends this knowledge by demonstrating the prognostic value of specific radiomic features derived from imaging data. The features we selected, such as statistical skewness, morphological elongation and GLSZM, offer quantitative information about tissue composition and morphology that may not be readily apparent from traditional imaging analysis. By leveraging radiomics techniques, we aim to complement existing knowledge and potentially uncover additional insights that may not be discernible through conventional methods alone.

The limitations of this study are its retrospective nature and a class imbalance due to the smaller number of events. We aimed to mitigate this problem by performing internal CV on the training data for feature selection. A three‐fold CV approach was used and repeated 10 times, ensuring that each fold contained sufficient events for training and validation and that the finally considered average model performance was sufficiently robust. Clearly, additional research is needed to validate our findings in independent cohorts and optimize the predictive models used for predicting short‐term mortality in APE.

## Conclusions

Radiomics parameters of the skeletal musculature and IMAT derived from CT images predict 30‐day mortality in APE but do not effectively predict 7‐day mortality.

## Conflict of interest statement

Our department of radiology maintains research cooperation with Siemens Healthineers, Erlangen, Germany. J.R. Kroeger received honoraria for scientific lectures from GE Healthcare and honoraria for clinical advisory board membership from Siemens Healthineers. J. Borggrefe received honoraria for scientific lectures from Philips Healthcare and Siemens Healthineers. I. Shahzadi and L. Johann Frohwein are employees of Siemens Healthineers. A. Zwanenburg, D. Schramm, H.J. Meyer, M. Hinnerichs, C. Moenninghoff, J.H. Niehoff and A. Surov declare no conflict of interest.

## Supporting information


**Table S1:** Image acquisition parameters of CTPA data.
**Figure S1:** An example of segmentation masks of skeletal muscle tissue (SM), intramuscular adipose tissue (IMAT), and both segmentations combined (SM + IMAT). Radiomics analysis was performed for each of these segmentation regions for the prediction of both 7‐day and 30‐day mortality.
**Table S2:** Image preprocessing parameters for CTPA data. All calculations were performed in 2D. Configuration file for feature extraction can be accessed here: https://github.com/shahzadir/Acute‐pulmonary‐embolismradiomic.
**Table S3:** Median AUC for 30‐day mortality prognosis in PE using features based on CTPA using crossvalidation of the training data. SM + IMAT features with an occurrence ≥50% are shown here. Features with a repeated occurrence across 2 out of 3 of the feature selection methods are presented in bold. AUC: area under the curve, CV: cross‐validation, CT: computed tomography, UR: univariate logistic regression, MRMR: minimum redundancy maximum relevance, MIM: mutual information maximization.
**Figure S2:** Correlation plot of features with occurrence > 50% in CV folds for each feature selection method.
**Table S4:** Median area under curve (AUC) of models based on different feature‐selection methods and classifiers for the prognosis of 30‐day all‐cause mortality in acute pulmonary embolism (APE) for skeletal muscle (SM), intramuscular adipose tissue (IMAT), and both tissues combined (SM + IMAT). Average AUC values across 3 feature selection method are shown for (a) training and (b) validation models of 10 times repeated 3‐fold cross‐validation (CV). Overall, gradient‐boosted linear models (XGB_lm) showed higher performance for all feature selection methods compared to multivariable logistic regression (Glm_logistic) and random forest (RF) classifiers. The results from random forest model showed overfitting compared to other learners.
**Figure S3:** Each learner's performance across each feature selection method for predicting 30‐day all‐cause mortality in PE patients. The models were built on features extracted from skeletal muscle and intramuscular adipose tissue (SM + IMAT). Median area under curve (AUC) of models based on different feature‐selection methods and classifiers for the prognosis of 30‐day all‐cause mortality in acute pulmonary embolism (APE). AUC values are shown for (a) training and (b) validation models of 10 times repeated 3‐fold cross‐validation (CV). Overall, gradient boosted linear models (XGB_lm) showed higher performance for all feature selection methods compared to multivariable logistic regression (Glm_logistic) and random forest (RF) classifiers. The average of the model performance across all feature selection method for SM + IMAT based models is shown previously in Table S4.
**Figure S4:** Box plot of Yeo‐Johnson transformed, and z‐score normalized features selected in best performing radiomics signature obtained from Skeletal muscle and intramuscular adipose tissue (SM + IMAT) in training data. PE status 1 indicates patients with survival ≤ 30 days and 0 indicates alive beyond 30 days.
**Table S5:** ROC‐AUC comparison of models with DeLong's test.
**Table S6:** Final models for the 30‐day all‐cause mortality prediction in acute pulmonary embolism (APE) patients based on radiomics signature based on combined ROI of skeletal muscle (SM) and intramuscular adipose tissue (IMAT) signature built using gradient boosted linear models (XGB_lm). In addition, transformation parameters from the Yeo‐Johnson transformation and z‐normalization estimates are given.
**Figure S5:** Calibration plots on training and test data for prediction of 30‐day all‐cause mortality in patients with acute pulmonary embolism (APE) resulting from best performing (a) clinical signature (sPESI), and (b) radiomics signature based on combined skeletal muscle and intramuscular adipose tissue (SM + IMAT). For calibration, data (thick lines) and 95% confidence intervals (shaded regions) are shown together with linear regression lines (solid lines) that follow the optimal expectation (dashed lines). Density of expected probabilities is shown above the calibration plot. Since most APE patients survived beyond 30‐days, the majority of predicted probabilities are close to 0, leading to clustering of points near the bottom of the plot.

## References

[1] Goldhaber SZ , Visani L , De Rosa M . Acute pulmonary embolism: clinical outcomes in the International Cooperative Pulmonary Embolism Registry (ICOPER). Lancet 1999;353:1386–1389. 10227218 10.1016/s0140-6736(98)07534-5

[2] Ng ACC , Chung T , Sze Chiang Yong A , et al. Long‐term cardiovascular and noncardiovascular mortality of 1023 patients with confirmed acute pulmonary embolism. Cardiol Res Pract 2011;4:122–128. 10.1161/CIRCOUTCOMES.110.958397 21098781

[3] Aujesky D , Obrosky DS , Stone RA , Auble TE , Perrier A , Cornuz J , et al. Derivation and validation of a prognostic model for pulmonary embolism. Am J Respir Crit Care Med 2005;172:1041–1046. 16020800 10.1164/rccm.200506-862OC PMC2718410

[4] Zondag W , Mos ICM , Creemers‐Schild D , et al. Outpatient treatment in patients with acute pulmonary embolism: the Hestia Study. J Thromb Haemost 2011;9:1500–1507. 21645235 10.1111/j.1538-7836.2011.04388.x

[5] Palas M , Silva BV , Jorge C , Almeida AG , Pinto FJ , Caldeira D . The accuracy of Hestia and simplified PESI to predict the prognosis in pulmonary embolism: systematic review with meta‐analysis. TH Open 2022;6:e347–e353. 36452203 10.1055/a-1942-2526 PMC9593482

[6] Gupta R , Fortman DD , Morgenstern DR , Cooper CJ . Short‐ and long‐term mortality risk after acute pulmonary embolism. Clin Cardiol 2018;20:30311090. 10.1007/s11886-018-1084-6 30311090

[7] Meinel FG , Nance JW Jr , Schoepf UJ , Hoffmann VS , Thierfelder KM , Costello P , et al. Predictive value of computed tomography in acute pulmonary embolism: systematic review and meta‐analysis. Am J Med 2015;128:747–759. 25680885 10.1016/j.amjmed.2015.01.023

[8] Kizilarslanoglu MC , Kuyumcu ME , Yesil Y , Halil M . Sarcopenia in critically ill patients. J Anesth 2016;30:884–890. 27376823 10.1007/s00540-016-2211-4

[9] Simpson G , Wilson J , Vimalachandran D , McNicol F , Magee C . Sarcopenia estimation using psoas major enhances P‐POSSUM mortality prediction in older patients undergoing emergency laparotomy: cross‐sectional study. Eur J Trauma Emerg Surg 2021;1–10. 33884449 10.1007/s00068-021-01669-1

[10] Meyer HJ , Wienke A , Surov A . Computed tomography‐defined body composition as prognostic markers for unfavorable outcomes and in‐hospital mortality in coronavirus disease 2019. J Cachexia Sarcopenia Muscle 2022;13:159–168. 35018725 10.1002/jcsm.12868 PMC8818651

[11] Meyer HJ , Benkert F , Bailis N , Lerche M , Denecke T , Surov A . Low skeletal muscle mass defined by thoracic CT as a prognostic marker in acute pulmonary embolism. Eur J Radiol 2022;98:111622. 10.1016/j.nut.2022.111622 35436690

[12] Liu Z , Wang S , Dong D , Wei J , Fang C , Zhou X , et al. The applications of radiomics in precision diagnosis and treatment of oncology: opportunities and challenges. Theranostics 2019;9:1303–1322. 30867832 10.7150/thno.30309 PMC6401507

[13] Lubner MG , Smith AD , Sandrasegaran K , Sahani DV , Pickhardt PJ . CT texture analysis: definitions, applications, biologic correlates, and challenges. Radiographics 2017;37:1483–1503. 28898189 10.1148/rg.2017170056

[14] Leonhardi J , Bailis N , Lerche M , Denecke T , Surov A , Meyer HJ . Computed tomography embolus texture analysis as a prognostic marker of acute pulmonary embolism. J Am Coll Radiol 2023;74:461–471. 10.1177/00033197221111862 PMC10070556 35973807

[15] Zwanenburg A , Leger S , Agolli L , Pilz K , Troost EGC , Richter C , et al. Assessing robustness of radiomic features by image perturbation. Sci Rep 2019;9:614. 30679599 10.1038/s41598-018-36938-4 PMC6345842

[16] Zwanenburg A , Vallières M , Abdalah MA , Aerts HJWL , Andrearczyk V , Apte A , et al. The image biomarker standardization initiative: standardized quantitative radiomics for high‐throughput image‐based phenotyping. Radiology 2020;295:328–338. 32154773 10.1148/radiol.2020191145 PMC7193906

[17] Zwanenburg A . Standardisation and harmonisation efforts in quantitative imaging. Eur Radiol 2023;33:8842–8843. 37466706 10.1007/s00330-023-09921-5

[18] Zwanenburg A , Löck S . familiar: end‐to‐end automated machine learning and model evaluation. 2021. Available from: https://github.com/alexzwanenburg/familiar

[19] Peng H , Long F , Ding C . Feature selection based on mutual information criteria of max‐dependency, max‐relevance, and min‐redundancy. IEEE Trans Pattern Anal Mach Intell 2005;27:1226–1238. 16119262 10.1109/TPAMI.2005.159

[20] Gel'fand IM , Yaglom AM . Computation of the amount of information about a stochastic function contained in another such function. Am Math Soc Transl 1957;12:3–52.

[21] Cox DR . The regression analysis of binary sequences. J R Stat Soc B Methodol 1958;20:215–232.

[22] Chen T , Guestrin C . Xgboost: a scalable tree boosting system. In Proceedings of the 22nd ACM SIGKDD International Conference on Knowledge Discovery and Data Mining; 2016. p 785–794.

[23] Breiman L . Bagging predictors. Mach Learn 1996;24:123–140.

[24] Hothorn T , Lausen B . On the exact distribution of maximally selected rank statistics. Comput Stat Data Anal 2003;43:121–137.

[25] Efron B , Hastie T . Computer age statistical inference. Cambridge University Press; 2016.

[26] Hosmer DW , Lemesbow S . Goodness of fit tests for the multiple logistic regression model. Commun Stat ‐ Theory Methods 1980;9:1043–1069.

[27] Konstantinides SV , Meyer G . The 2019 ESC guidelines on the diagnosis and management of acute pulmonary embolism. Eur Heart J 2019;40:3453–3455. 31697840 10.1093/eurheartj/ehz726

[28] Zhou XY , Ben SQ , Chen HL , Ni SS . The prognostic value of pulmonary embolism severity index in acute pulmonary embolism: a meta‐analysis. Respir Res 2012;13:111. 23210843 10.1186/1465-9921-13-111 PMC3571977

[29] Nithianandan H , Reilly A , Tritschler T , Wells PS . Applying rigorous eligibility criteria to studies evaluating prognostic utility of serum biomarkers in pulmonary embolism: a systematic review and meta‐analysis. Thromb Res 2020;195:195–208. 32745746 10.1016/j.thromres.2020.07.037

[30] Söhne M , ten Wolde M , Boomsma F , et al. Brain natriuretic peptide in hemodynamically stable acute pulmonary embolism. J Thromb Haemost 2006;4:552–556. 16405522 10.1111/j.1538-7836.2005.01752.x

[31] Lankeit M , Jiménez D , Kostrubiec M , et al. Validation of N‐terminal pro‐brain natriuretic peptide cut‐off values for risk stratification of pulmonary embolism. J Thromb Haemost 2014;43:1669–1677. 10.1183/09031936.00211613 24627529

[32] Wiliński J , Skwarek A , Borek R , Chukwu O , Ciuk K , Stolarz‐Skrzypek K , et al. Subcostal echocardiographic assessment of tricuspid annular kick (SEATAK): a novel independent predictor of 30‐day mortality in patients with acute pulmonary embolism. J Am Soc Echocardiogr 2022;80:1127–1135. 10.33963/KP.a2022.0213 36088580

[33] Foley RW , Glenn‐Cox S , Rossdale J , Mynott G , Burnett TA , Brown WJH , et al. Automated calculation of the right ventricle to left ventricle ratio on CT for the risk stratification of patients with acute pulmonary embolism. J Cardiovasc Comput Tomogr 2021;31:6013–6020. 10.1007/s00330-020-07605-y 33459854

[34] Han DS , Chang KV , Li CM , Lin YH , Kao TW , Tsai KS , et al. Skeletal muscle mass adjusted by height correlated better with muscular functions than that adjusted by body weight in defining sarcopenia. Sci Rep 2016;6:19457. 26785759 10.1038/srep19457 PMC4726295

[35] Chang KV , Chen JD , Wu WT , Huang KC , Hsu CT , Han DS . Association between loss of skeletal muscle mass and mortality and tumor recurrence in hepatocellular carcinoma: a systematic review and meta‐analysis. Liver Cancer 2018;7:90–103. 29662836 10.1159/000484950 PMC5892377

[36] Mitsiopoulos N , Baumgartner RN , Heymsfield SB , Lyons W , Gallagher D , Ross R . Cadaver validation of skeletal muscle measurement by magnetic resonance imaging and computerized tomography. J Appl Physiol 1998;85:115–122. 9655763 10.1152/jappl.1998.85.1.115

[37] Saalfeld S , Kreher R , Hille G , Niemann U , Hinnerichs M , Öcal O , et al. Prognostic role of radiomics‐based body composition analysis for the 1‐year survival for hepatocellular carcinoma patients. J Cachexia Sarcopenia Muscle 2023;14:2301–2309. 37592827 10.1002/jcsm.13315 PMC10570090

[38] Addison O , Marcus RL , LaStayo PC , Ryan AS . Intermuscular fat: a review of the consequences and causes. Int J Endocrinol 2014. 10.1155/2014/309570 PMC3910392 24527032

[39] Liao Q , Qu S , Tang LX , Li LP , He DF , Zeng CY , et al. Irisin exerts a therapeutic effect against myocardial infarction via promoting angiogenesis. Acta Pharmacol Sin 2019;40:1314–1321. 31061533 10.1038/s41401-019-0230-z PMC6786355

[40] Harris MP , Zeng S , Zhu Z , Lira VA , Yu L , Hodgson‐Zingman DM , et al. Myokine musclin is critical for exercise‐induced cardiac conditioning. Int J Mol Sci 2023;24:6525. 37047496 10.3390/ijms24076525 PMC10095193

[41] Szaroszyk M , Kattih B , Martin‐Garrido A , Trogisch FA , Dittrich GM , Grund A , et al. Skeletal muscle derived Musclin protects the heart during pathological overload. Nat Commun 2022;13:149. 35013221 10.1038/s41467-021-27634-5 PMC8748430

[42] Otaka N , Shibata R , Ohashi K , Uemura Y , Kambara T , Enomoto T , et al. Myonectin is an exercise‐induced myokine that protects the heart from ischemia‐reperfusion injury. Circ Res 2018;123:1326–1338. 30566056 10.1161/CIRCRESAHA.118.313777

[43] Szabó MR , Pipicz M , Csont T , Csonka C . Modulatory effect of myokines on reactive oxygen species in ischemia/reperfusion. Int J Mol Sci 2020;21:9382. 33317180 10.3390/ijms21249382 PMC7763329

[44] Shibata A , Hanatani A , Izumi Y , Kitada R , Iwata S , Yoshiyama M . Serum brain‐derived neurotrophic factor level and exercise tolerance complement each other in predicting the prognosis of patients with heart failure. Heart Vessels 2018;33:1325–1333. 29700574 10.1007/s00380-018-1174-9

[45] Bhullar AS , Anoveros‐Barrera A , Dunichand‐Hoedl A , Martins K , Bigam D , Khadaroo RG , et al. Lipid is heterogeneously distributed in muscle and associates with low radiodensity in cancer patients. J Cachexia Sarcopenia Muscle 2020;11:735–747. 31989803 10.1002/jcsm.12533 PMC7296261

[46] Meyer HJ , Kardas H , Schramm D , Bär C , Wienke A , Borggrefe J , et al. CT‐defined pectoralis muscle mass and muscle density are associated with mortality in acute pulmonary embolism. A multicenter analysis. Clin Nutr 2023;42:1036–1040. 37156143 10.1016/j.clnu.2023.04.022

