# Supplementary material for: Short‐term mortality prediction in acute pulmonary embolism: Radiomics values of skeletal muscle and intramuscular adipose tissue
Source: J Cachexia Sarcopenia Muscle. 2024 Jun 10;15(4):1430–40. doi: 10.1002/jcsm.13488 (PMC11294025; doi:10.1002/jcsm.13488)
Supplement: Supplementary file 1 — Table S1: Image acquisition parameters of CTPA data. Figure S1: An example of segmentation masks of skeletal muscle tissue (SM), intramuscular adipose tissue (IMAT), and both segmentations combined (SM + IMAT). Radiomics analysis was performed for each of these segmentation regions for the prediction of both 7‐day and 30‐day mortality. Table S2: Image preprocessing parameters for CTPA data. All calculations were performed in 2D. Configuration file for feature extraction can be accessed here: https://github.com/shahzadir/Acute‐pulmonary‐embolismradiomic. Table S3: Median AUC for 30‐day mortality prognosis in PE using features based on CTPA using crossvalidation of the training data. SM + IMAT features with an occurrence ≥50% are shown here. Features with a repeated occurrence across 2 out of 3 of the feature selection methods are presented in bold. AUC: area under the curve, CV: cross‐validation, CT: computed tomography, UR: univariate logistic regression, MRMR: minimum redundancy maximum relevance, MIM: mutual information maximization. Figure S2: Correlation plot of features with occurrence > 50% in CV folds for each feature selection method. Table S4: Median area under curve (AUC) of models based on different feature‐selection methods and classifiers for the prognosis of 30‐day all‐cause mortality in acute pulmonary embolism (APE) for skeletal muscle (SM), intramuscular adipose tissue (IMAT), and both tissues combined (SM + IMAT). Average AUC values across 3 feature selection method are shown for (a) training and (b) validation models of 10 times repeated 3‐fold cross‐validation (CV). Overall, gradient‐boosted linear models (XGB_lm) showed higher performance for all feature selection methods compared to multivariable logistic regression (Glm_logistic) and random forest (RF) classifiers. The results from random forest model showed overfitting compared to other learners. Figure S3: Each learner's performance across each feature selection method for predicting 30‐day [file JCSM-15-1430-s001.pdf]

Short-term Mortality Prediction in Acute Pulmonary Embolism: Radiomics Values of Skeletal Muscle & Intramuscular Adipose Tissue

Table S1: Image acquisition parameters of CTPA data

**Imaging Parameters**	**Center 1 (450)**	**Center 2 (210)**	**Center 3 (169)**
**Voxel spacing (mm)**			
0.5/0.6/0.7/0.8/0.9/1.0	3/34/163/203/37/10	1/18/93/68/22/8	3/33/117/15/1/0
**Slice thickness (mm)**			
0.5/1/2/3/4/5/6/7	0/450/0/0/0/0/0/0	0/0/91/115/0/4/0/0	1/0/45/48/0/74/0/1
**Reconstruction kernel**			
B/B20f/B26f/B30f/B31s/<br>B31f/B40f/B41s/B41f/B70f<br>/I26f/I30f/I31f/FC08/FC13/<br>FC52	0/0/152/0/0/<br>30/0/0/0/0<br>/21/7/5/228/7/<br>0	199/0/0/0/1<br>/7/1/1/0/0<br>/0/0/1/0/0/<br>0	0/1/0/0/0/<br>0/1/1/29/64<br>/0/0/0/0/34/<br>40
**Tube Voltage (kV)**			
80/100/110/120/140	4/273/0/172/1	4/71/2/133/0	0/0/0/167/2
**Manufacturer**			
SIEMENS/TOSHIBA/PHILIP<br>S	215/235/0	8/0/202	95/74/0

**Image**	**Skeletal muscle (SM)**	**Intramuscular adipose tissue (IMAT)**	**Combined (SM+IMAT)**

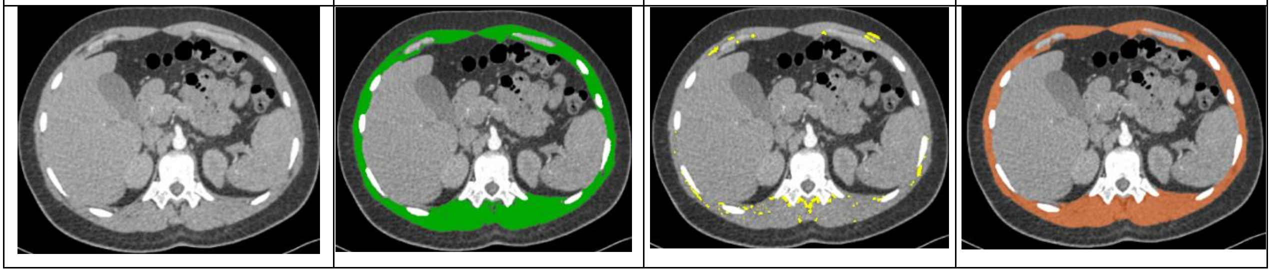

Figure S1: An example of segmentation masks of skeletal muscle tissue (SM), intramuscular adipose tissue (IMAT), and both segmentations combined (SM+IMAT). Radiomics analysis was performed for each of these segmentation regions for the prediction of both 7-day and 30-day mortality.

Table S2: Image preprocessing parameters for CTPA data. All calculations were performed in 2D. Configuration file for feature extraction can be accessed here: https://github.com/shahzadir/Acute-pulmonary-embolism-radiomics

**Parameters**	**Value**
Pre-interpolation filter	None
Intensity normalization	None
Interpolated isotropic voxel spacing (mm)	1
Image interpolation method	cubic
ROI interpolation method	linear
Re-segmentation range	[-110,150]
Merge method for texture matrices	slice merge
Discretisation method: fixed bin number (bins)	24

Section 1: Ranking scheme for feature selection

Here we explain an example of feature selection for 30-day mortality prediction in PE using features extracted from ROI comprising of skeletal muscle (SM) and intramuscular adipose tissue (IMAT) combined (SM+IMAT). 45 CTPA features with the highest mutual information with 30-day mortality were selected after hierarchical clustering. These features were then used to build a prognostic model. Feature selection and model building with internal validation was first performed within 10 repetitions of 3-fold cross-validation (CV) nested in the training dataset to identify an optimal signature. Three supervised feature-selection algorithms were considered: minimal redundancy maximum relevance (MRMR), mutual information maximization (MIM), and univariate logistic regression (LR). To avoid potential overfitting, only the five most relevant features were selected in each cross-validation fold. These features were then used to build a logistic regression (GLM_logistic), gradient-boosted linear model (XGB_lm), and random forest (RF) model on the internal training part and validated on the internal validation part. For each of the above-mentioned feature selection methods, the occurrence of every feature in the 30 modelling steps was counted and features were ranked according to their occurrences across the cross-validation folds. Table S3 shows features that were incorporated in XGB_lm models with ≥50 % occurrence in internal CV for each feature selection method. Finally, features that showed repeated occurrences across at least 2 out of 3 feature selection methods were selected (stat_skew, ih_mode_fbn_n24, szm_sze_2d_fbn_n24, morph_pca_elongation). Among these 4 selected features, ih_mode_fbn_n24 showed Spearman correlation $|\rho| > 0.5$ with stat_skew on the entire training data as shown in Figure S2. Therefore ih_mode_fbn_n24 was dropped due to its overall lower score. Finally, 3 features (stat_skew, szm_sze_2d_fbn_n24, morph_pca_elongation) were used to form. A model with this signature was then fitted on the entire training data and the trained model was applied to the held-out test data.

Table S3: Median AUC for 30-day mortality prognosis in PE using features based on CTPA using cross-validation of the training data. SM+IMAT features with an occurrence ≥50% are shown here. Features with a repeated occurrence across 2 out of 3 of the feature selection methods are presented in bold. AUC: area under the curve, CV: cross-validation, CT: computed tomography, UR: univariate logistic regression, MRMR: minimum redundancy maximum relevance, MIM: mutual information maximization.

<table>
<tr><th>Feature selection</th><th>CV training AUC</th><th>CV Validation AUC</th><th>Features</th><th>Occurrence</th><th>Selected features</th></tr>
<tr><td>MRMR</td><td>0.72</td><td>0.72</td><td>stat_skew</td><td>90</td><td rowspan="3">stat_skew
ih_mode_fbn_n24
szm_sze_2d_fbn_n24
morph_pca_elongation
Remarks: The above 4 features occurred in at least 2 out of 3 feature selection methods. ih_mode_fbn_n24 and szm_sze_2d_fbn_n24 showed a high correlation, so ih_mode_fbn_n24 was dropped and the remaining 3 features were considered for the signature</td></tr>
<tr><td>MIM</td><td>0.74</td><td>0.72</td><td>stat_skew
ih_mode_fbn_n24
szm_sze_2d_fbn_n24
morph_pca_elongation</td><td>100
77
67
53</td></tr>
<tr><td>UR</td><td>0.74</td><td>0.72</td><td>stat_skew
ih_mode_fbn_n24
cm_clust_shade_d1_2d_s_mrg_fbn_n24
szm_sze_2d_fbn_n24
morph_pca_elongation</td><td>100
93
90
63
57</td></tr>
</table>

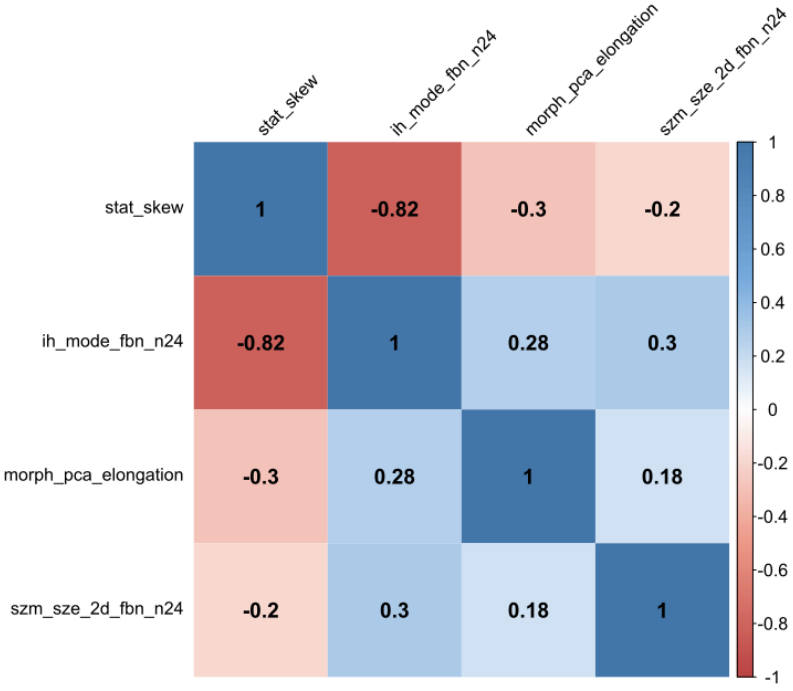

Figure S2: Correlation plot of features with occurrence > 50% in CV folds for each feature selection method.

Table S4: Median area under curve (AUC) of models based on different feature-selection methods and classifiers for the prognosis of 30-day all-cause mortality in acute pulmonary embolism (APE) for skeletal muscle (SM), intramuscular adipose tissue (IMAT), and both tissues combined (SM+IMAT). Average AUC values across 3 feature selection method are shown for (a) training and (b) validation models of 10 times repeated 3-fold cross-validation (CV). Overall, gradient-boosted linear models (XGB_lm) showed higher performance for all feature selection methods compared to multivariable logistic regression (Glm_logistic) and random forest (RF) classifiers. The results from random forest model showed overfitting compared to other learners.

**ROI**	**Learner**	**CV Training AUC 7-day**	**CV Validation AUC 7-day**	**CV Training AUC 30-day**	**CV Validation AUC 30-day**
**SM**	Glm_logistic	0.64	0.59	0.69	0.67
	RF	0.83	0.59	0.78	0.66
	**XGB_lm**	**0.66**	**0.60**	**0.69**	**0.68**
**IMAT**	Glm_logistic	0.64	0.56	0.66	0.59
	RF	0.82	0.56	0.76	0.59
	**XGB_lm**	**0.65**	**0.55**	**0.65**	**0.59**
**SM+IMAT**	Glm_logistic	0.69	0.62	0.72	0.70
	RF	0.81	0.59	0.80	0.69
	**XGB_lm**	**0.68**	**0.63**	**0.73**	**0.72**

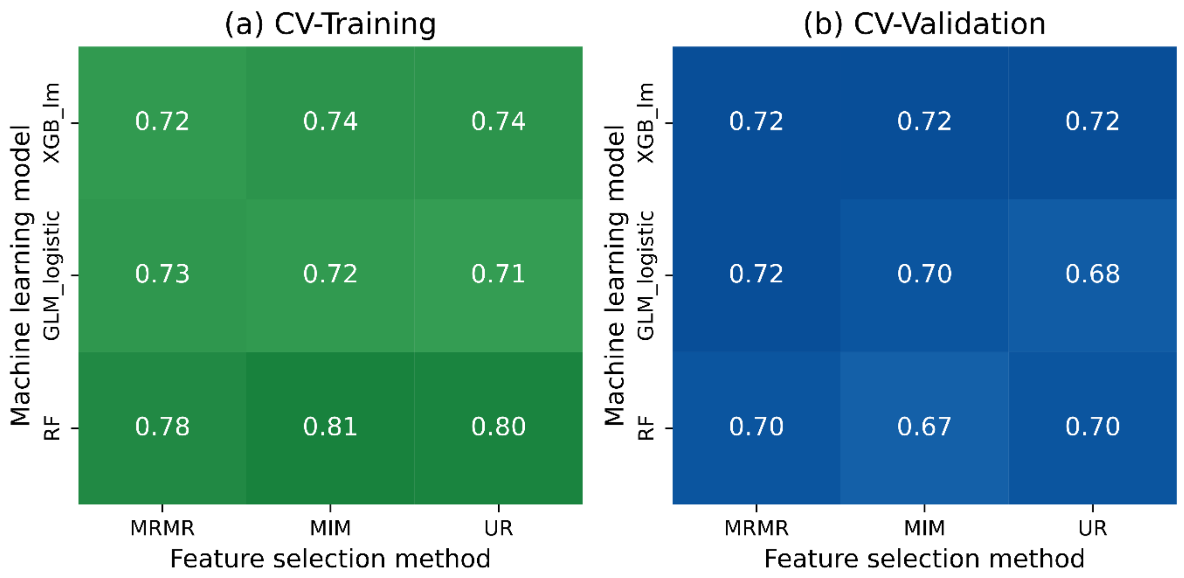

Figure S3: Each learner's performance across each feature selection method for predicting 30-day all-cause mortality in PE patients. The models were built on features extracted from skeletal muscle and intramuscular adipose tissue (SM+IMAT). Median area under curve (AUC) of models based on different feature-selection methods and classifiers for the prognosis of 30-day all-cause mortality in acute pulmonary embolism (APE). AUC values are shown for (a) training and (b) validation models of 10 times repeated 3-fold cross-validation (CV). Overall, gradient boosted linear models (XGB_lm) showed higher performance for all feature selection methods compared to multivariable logistic regression (Glm_logistic) and random forest (RF) classifiers. The average of the model performance across all feature selection method for SM+IMAT based models is shown previously in Table S4.

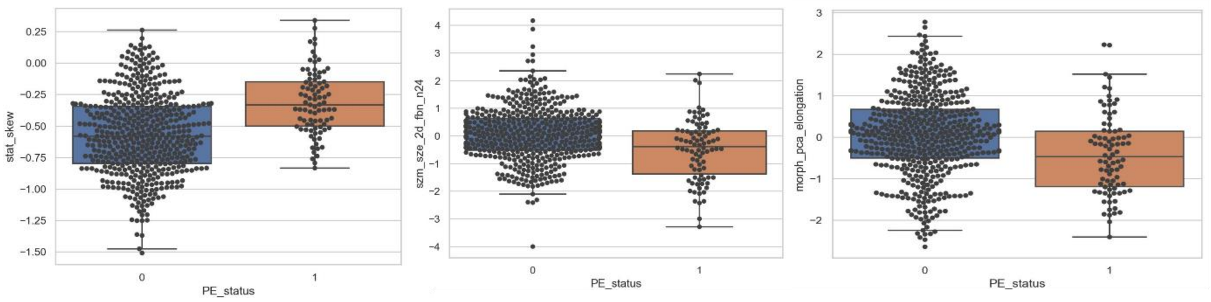

Figure S4: Box plot of Yeo-Johnson transformed, and z-score normalized features selected in best performing radiomics signature obtained from Skeletal muscle and intramuscular adipose tissue (SM+IMAT) in training data. PE status 1 indicates patients with survival ≤ 30 days and 0 indicates alive beyond 30 days.

Table S5: ROC-AUC comparison of models with DeLong's test.

Model	p-value train 30-day	p-value test 30-day	p-value train 7-day	p-value test 7-day
sPESI vs SM+IMAT	0.19	0.49	0.81	0.03
sPESI vs SM	0.72	0.12	0.53	0.05
sPESI vs IMAT	0.69	0.29	0.34	0.09
SM+IMAT vs SM	0.31	0.06	0.54	0.82
SM+IMAT vs IMAT	0.47	0.51	0.30	0.61
SM vs IMAT	0.93	0.38	0.77	0.60

Table S6: Final models for the 30-day all-cause mortality prediction in acute pulmonary embolism (APE) patients based on radiomics signature based on combined ROI of skeletal muscle (SM) and intramuscular adipose tissue (IMAT) signature built using gradient boosted linear models (XGB_lm). In addition, transformation parameters from the Yeo-Johnson transformation and z-normalization estimates are given.

<table>
<tr><th>Model</th><th>Feature</th><th>Yeo-Johnson (λ)</th><th>z-score normalization (mean, sigma)</th><th>XGB_lm model parmeters</th></tr>
<tr><td rowspan="3">Radiomics SM+IMAT</td><td>stat_skew</td><td>-</td><td>(-0.53,0.34)</td><td rowspan="3">n_boost: 0.76
learning rate: -1
lambda: -6
alpha: -3
sample_weighting: inverse_number_of_samples
sample_weighting_beta: -2</td></tr>
<tr><td>szm_sze_2d_fbn_n24</td><td>9.6</td><td>(10.12,1.62)</td></tr>
<tr><td>morph_pca_elongation</td><td>7.0</td><td>(10.19,2.87)</td></tr>
</table>

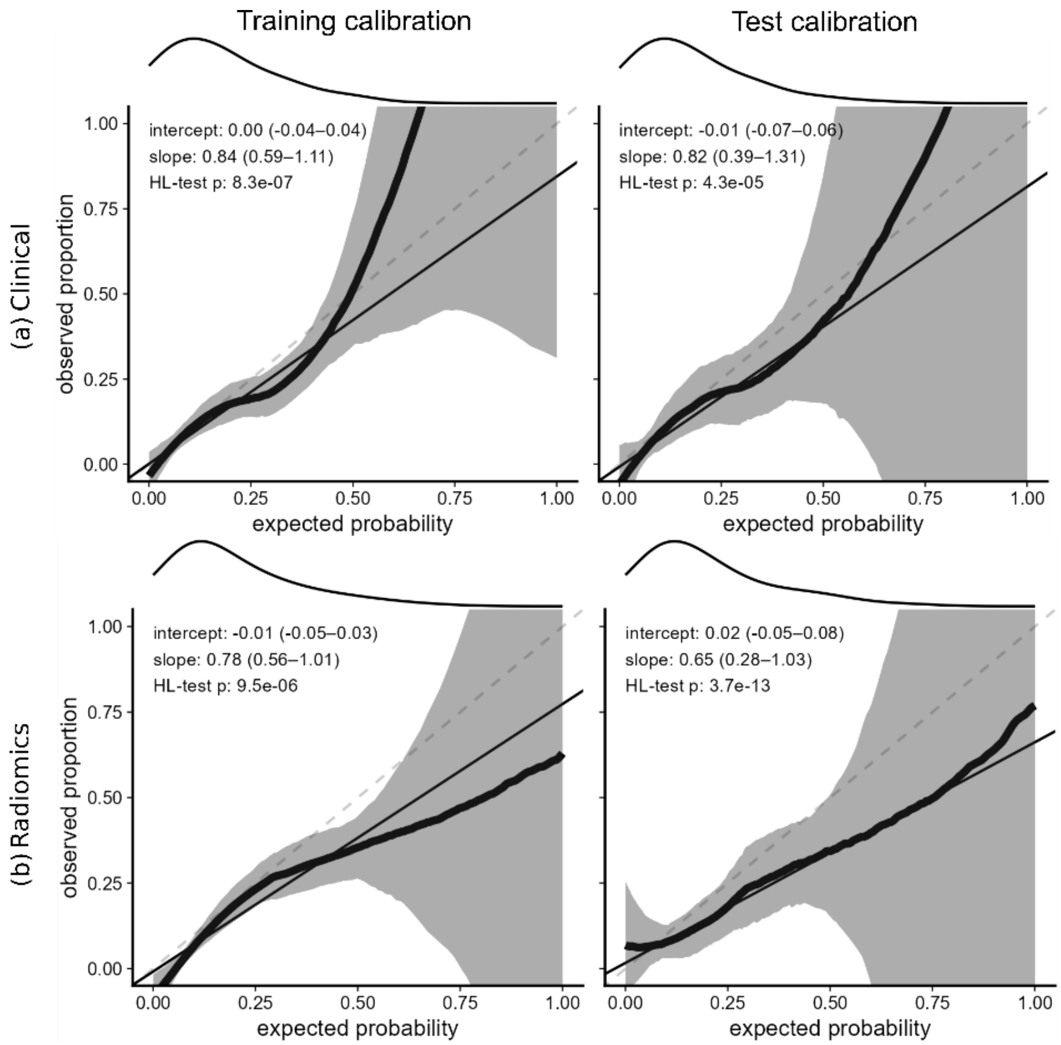

Figure S5: Calibration plots on training and test data for prediction of 30-day all-cause mortality in patients with acute pulmonary embolism (APE) resulting from best performing (a) clinical signature (sPESI), and (b) radiomics signature based on combined skeletal muscle and intramuscular adipose tissue (SM+IMAT). For calibration, data (thick lines) and 95% confidence intervals (shaded regions) are shown together with linear regression lines (solid lines) that follow the optimal expectation (dashed lines). Density of expected probabilities is shown above the calibration plot. Since most APE patients survived beyond 30-days, the majority of predicted probabilities are close to 0, leading to clustering of points near the bottom of the plot.
